# Dynamic Histone Modification Patterns in Key Transcription Factor Genes During Porcine Adipogenesis

**DOI:** 10.3390/genes17050521

**Published:** 2026-04-28

**Authors:** Mehmet Onur Aksoy, Jakub Wozniak, Monika Stachowiak, Izabela Szczerbal

**Affiliations:** Department of Genetics and Animal Breeding, Poznan University of Life Sciences, 60-637 Poznan, Poland; onur.aksoy@up.poznan.pl (M.O.A.); jakub.wozniak@up.poznan.pl (J.W.); monika.dragan@up.poznan.pl (M.S.)

**Keywords:** adipocytes, ChIP-qPCR, histone marks, mesenchymal stem cells, pig

## Abstract

Background: Adipogenesis is governed by a complex interplay between transcriptional regulation and epigenetic remodeling. While many transcriptional pathways have been well characterized, less is known about how chromatin-level regulation shapes the timing of gene expression, particularly in large animal models such as pigs. In this study, we investigated histone modification patterns associated with four key adipogenic transcription factor genes—*PPARG*, *GATA2*, *CEBPA*, and *CEBPB*—in porcine mesenchymal stem cells (MSCs) undergoing adipogenic differentiation. Methods: Using RT-qPCR and ChIP-qPCR, we profiled gene transcription levels and epigenetic marks, including promoter- and exon-specific enrichment of the activating histone marks H3K9ac and H4K8ac, as well as the repressive mark H4K20me3, across six time points (day 0, 2, 4, 6, 8, and 10). Results: Although *PPARG* and *GATA2* are located in close proximity on porcine chromosome 13, they exhibited distinct histone modification profiles. *PPARG* showed progressive promoter acetylation (H4K8ac) accompanied by transcriptional activation, whereas *GATA2* displayed decreased exon acetylation (H3K9ac) associated with declining expression. In contrast, the H4K20me3 profile was similar for both genes, suggesting no direct association with their transcriptional activity. Interestingly, *CEBPA* (chromosome 6) and *CEBPB* (chromosome 17) exhibited temporally distinct histone modification patterns consistent with their roles in intermediate and early stages of adipogenic differentiation, respectively. Increased enrichment of the H3K9ac mark preceded the rise in transcript levels of the analyzed genes. Promoter regions showed higher enrichment of H4K8ac compared with exonic regions. A higher level of H4K20me3 was also observed for *CEBPA* and *CEBPB* than for *PPARG* and *GATA2*, which appeared to be more related to chromosomal localization than to direct transcriptional regulation. Conclusions: Together, these results reveal complex interactions between transcriptional dynamics and selected histone modifications that depend on both the gene analyzed and the stage of adipocyte differentiation. This study provides new insights into the epigenetic regulation of porcine adipogenesis and highlights chromatin context as an additional layer influencing transcriptional control.

## 1. Introduction

Adipogenesis, the process by which precursor cells differentiate into mature adipocytes, plays a crucial role in several biological processes, including energy homeostasis and lipid metabolism. This process is tightly regulated and involves a complex network of molecular pathways controlled by multiple regulatory elements [1]. Within this intricate regulation, numerous transcription factors and epigenetic regulators act in a coordinated manner, contributing to either the activation or repression of adipogenic gene expression. Among the key transcription factors that govern adipocyte differentiation, peroxisome proliferator-activated receptor gamma (PPARG), CCAAT/enhancer-binding proteins (CEBPA and CEBPB), and GATA Binding Protein 2 (GATA2) are well known for their critical roles in the initiation and progression of adipogenesis. While PPARG and members of the CEBP family promote the expression of pro-adipogenic genes, GATA2 is recognized as a negative regulator that inhibits adipogenic gene expression and thereby acts as a suppressor of adipocyte differentiation [2,3,4].

Recent studies have demonstrated that transcriptional regulation during adipogenesis is not solely governed by transcription factors but is also strongly influenced by epigenetic mechanisms, including chromatin structure and histone modifications [5,6,7,8]. Epigenetic marks such as histone acetylation and methylation dynamically change throughout the differentiation process, modulating the accessibility of chromatin and the transcriptional activity of key adipogenic regulators [9,10,11]. Histone acetylation marks associated with euchromatin, such as H3K9ac and H4K8ac, are generally linked to transcriptional activation and an open chromatin conformation; however, their presence does not necessarily indicate active transcription in all contexts, as they may also reflect a more permissive chromatin state or be involved in processes other than direct gene activation. In contrast, trimethylation marks like H4K20me3, H3K27me3 and H3K9me3 are strongly associated with heterochromatin, and correlated with transcriptional repression, gene silencing and chromatin compaction [8,12,13]. During adipocyte differentiation, chromatin undergoes substantial structural remodeling driven by dynamic changes in histone post-translational modifications. Repressive H4K20me3 is progressively enriched at genomic regions undergoing transcriptional silencing, supporting heterochromatin formation and reduced accessibility during lineage commitment [14]. In contrast, activating acetylation marks such as H3K9ac and H4K8ac promotes transcription initiation by loosening nucleosome packing and facilitating transcription factor binding to adipogenic regulatory elements [8,15]. These activating marks are particularly associated with enhanced expression of master regulators, including *PPARG* and *CEBPA*, as preadipocytes transition to a mature adipocyte state [8]. Importantly, studies in porcine mesenchymal stem cell-derived adipocytes have demonstrated that the balance between H4K20me3 accumulation and the elevation of H3K9ac/H4K8ac closely reflects stage-specific chromatin accessibility and activation of adipogenesis [10]. Together, these observations highlight that assessing both permissive and repressive histone signatures is essential for understanding transcriptional control mechanisms that drive adipogenesis. Understanding the distribution and correlation of these histone modifications between promoter and exon regions of adipogenic genes provides valuable insights into the epigenetic mechanisms that regulate this differentiation process.

Previous research has reported that histone modifications play a role in the activation of *PPARG* and *CEBPB*, whereas regulatory factors such as *GATA2* exhibit an opposing epigenetic profile, acting as suppressors of adipogenic differentiation [1,16,17]. Most of these findings originate from murine systems, particularly studies performed in the 3T3-L1 mouse preadipocyte cell line, where acetylation marks at *PPARG* and *CEBPB* promoters have been associated with enhanced adipogenic transcription, while repressive methylation marks at *GATA2* loci have been linked to suppression of adipocyte maturation [17]. Similar regulatory patterns have also been observed in human primary preadipocytes and murine models, supporting the idea that chromatin remodeling is essential for adipogenesis [1]. Broader epigenetic analyses in mammals, including human and murine studies, also indicate that histone-based regulation is a conserved feature of adipocyte differentiation [16]. Although the regulation of adipogenesis shares many conserved mechanisms across species, chromatin dynamics and epigenetic architecture can differ, especially in livestock animals. In pigs, adipose tissue biology is not only important for metabolic research but also directly linked to fat deposition and meat quality traits [18,19]. Moreover, pigs display strong metabolic and endocrine similarities to humans, making them highly informative models for investigating obesity, insulin resistance, and other metabolic disorders [20]. Their adipocyte development and lipid metabolism closely resemble human physiology, including preadipocyte maturation [21]. In our previous study, we examined the abundance and nuclear distribution of selected heterochromatin (H3K9me3, H3K27me3, and H4K20me3) and euchromatin (H4K8ac, H3K4me3, and H3K9ac) histone marks during in vitro adipogenesis [10]. However, that study focused primarily on global changes in histone mark distribution and did not address gene-specific regulatory regions. Therefore, examining promoter- and exon-specific histone modifications in porcine mesenchymal stem cells provides novel biological insight and helps bridge the existing knowledge gap between human, mouse, and livestock adipogenesis research.

In the present study, we aimed to investigate the relationships between histone modifications (H4K20me3, H4K8ac, and H3K9ac) within the promoter and exon regions of several key adipogenic genes (*PPARG*, *GATA2*, *CEBPA*, and *CEBPB*) during adipocyte differentiation from porcine mesenchymal stem cells. *PPARG* and *GATA2*, which are located on the same chromosome and exhibit antagonistic regulatory activity, were analyzed alongside *GP9*, a gene located on the same chromosome but not associated with adipogenesis, which served as a control. To address this objective, we performed ChIP-qPCR analyses targeting three major histone modifications. These epigenetic measurements were complemented by transcriptional profiling using real-time PCR, followed by correlation analysis to determine how chromatin signatures align with gene activation or repression throughout adipocyte maturation. Samples were collected at six defined time points (day 0, 2, 4, 6, 8, and 10). Understanding these relationships improves our knowledge of adipogenesis and provides insight into how chromatin changes influence transcriptional regulation during adipocyte differentiation.

## 2. Materials and Methods

### 2.1. Cell Culture and Adipogenesis Induction

Adipose-derived mesenchymal stem cells (AD-MSC) were isolated from adipose tissue collected post mortem from Large White pigs at a commercial slaughterhouse. As no experimental procedures were conducted on live animals, ethical approval was not required in accordance with applicable regulations. AD-MSC were cultured in Advanced DMEM medium (Gibco, Thermo Fisher Scientific, Waltham, MA, USA), supplemented with 10% FBS (Sigma-Aldrich, Darmstadt, Germany), 5 ng/mL FGF-2 (PromoKine, Heidelberg, Germany), 2 mM L-Glutamine (Gibco, Thermo Fisher Scientific, Waltham, MA, USA), 1 mM 2-mercaptoethanol (Sigma-Aldrich, Darmstadt, Germany), 1× antibiotic-antimycotic solution (Sigma-Aldrich, Darmstadt, Germany), and 1× MEM NEAA (Gibco, Thermo Fisher Scientific, Waltham, MA, USA). These cells were maintained at 37 °C in a 5% CO_2_ atmosphere. Once the cells reached confluency, adipogenesis was initiated using a differentiation medium. This differentiation medium consisted of Advanced DMEM (Gibco, Thermo Fisher Scientific, Waltham, MA, USA), 10% FBS (Sigma-Aldrich, Darmstadt, Germany), 1× antibiotic-antimycotic solution (Sigma-Aldrich, Darmstadt, Germany), 5 ng/mL FGF-2 (PromoKine, Heidelberg, Germany), 1× Linoleic Acid Albumin (Sigma-Aldrich, Darmstadt, Germany), 1× ITS Supplement (Sigma-Aldrich, Darmstadt, Germany), 1 µM Dexamethasone (Sigma-Aldrich, Darmstadt, Germany), 100 µM Indomethacin (Sigma-Aldrich, Darmstadt, Germany), and 50 µM IBMX (Sigma-Aldrich, Darmstadt, Germany). The differentiation process was conducted for ten days, with the medium being changed every 24 h. The progression of adipogenesis was monitored by observing lipid droplet formation under phase-contrast microscopy (TS100 Eclipse, Nikon, Melville, NY, USA) and by BODIPY (Life Technologies, Grand Island, NY, USA) staining, following the protocol described previously by Aksoy et al. [22].

### 2.2. RNA Isolation, cDNA Synthesis, and Real-Time PCR

Total RNA was isolated from AD-MSC cell cultures and differentiating cells utilizing the TriPure Isolation Reagent (Roche Diagnostics, Mannheim, Germany). Following thorough quantitative and qualitative assessments with a Nanodrop 2000 spectrophotometer (Thermo Fisher Scientific, Waltham, MA, USA), one microgram of RNA was then converted into complementary DNA (cDNA) using a Transcriptor First Strand cDNA Synthesis Kit (Roche, Basel, Germany). For real-time PCR, specific primers were designed for each target transcript. These primers were designed to bind to distinct exons, referencing the Sscrofa 11.1 sequence (details in Table S1). Primer efficiency was determined based on standard curves, and specificity was confirmed by melt curve analysis. All PCR reactions were conducted in triplicate on a LightCycler 480 II instrument (Roche, Basel, Germany) employing the LightCycler 480 SYBR Green I Master Kit (Roche, Basel, Germany). The relative messenger RNA (mRNA) levels of the genes under investigation were determined via the second derivative maximum method. Finally, the obtained results were normalized against the geometric mean expression of the reference genes, *RPL27* and *PPIA.*

### 2.3. ChIP-qPCR Analysis

Chromatin immunoprecipitation followed by quantitative PCR (ChIP-qPCR) was performed to characterize histone modification enrichment at promoter and exon regions of adipogenic genes throughout differentiation. The procedure was carried out using the iDeal ChIP-seq Kit for Histones (Diagenode, Liège, Belgium; Kit Cat# C01010051) according to the manufacturer’s instructions, with minor adjustments optimized for porcine mesenchymal stem cells (AD-MSC). Cells were harvested at six differentiation time points (days 0, 2, 4, 6, 8, and 10), and culture medium was replaced daily to maintain consistent adipogenic induction. Cells were cross-linked with 1% formaldehyde for 8 min at room temperature, followed by quenching with 0.125 M glycine for 5 min. After two washes with ice-cold PBS, cells were scraped and lysed using SDS Lysis Buffer. Chromatin was sheared to ~100–500 bp fragments by sonication using a Bioruptor (Diagenode, Liège, Belgium). Following centrifugation at 14,000× *g* for 10 min at 4 °C, the soluble chromatin fraction was collected for immunoprecipitation. Immunoprecipitation was performed overnight at 4 °C using antibodies against H4K20me3 (Rabbit polyclonal, Abcam, Cambridge, UK; Cat# ab9053), H4K8ac (Rabbit polyclonal, Abcam, Cambridge, UK; Cat# ab15823), and H3K9ac (Rabbit polyclonal, Abcam, Cambridge, UK; Cat# ab10812) (Table S2). Rabbit IgG provided within the kit was used as a negative control. Immunocomplexes were retrieved using Protein A magnetic beads, washed sequentially to remove nonspecific binding, and eluted with the Elution Buffer supplied in the kit. Cross-links were reversed at 65 °C overnight and DNA was purified using silica-based spin columns. Quantitative PCR was performed using the LightCycler 480 II instrument (Roche, Basel, Germany) and the LightCycler 480 SYBR Green I Master Kit (Roche, Basel, Germany). Primers were designed to specifically amplify promoter and exon regions of *PPARG, GATA2, GP9, CEBPA,* and *CEBPB* (Table S1). Each 20 µL reaction contained 1 µL of immunoprecipitated DNA and was run in technical replicates. PCR conditions were as follows: initial denaturation at 95 °C for 5 min, followed by 40 cycles of 95 °C for 10 s and 60 °C for 30 s. Melting-curve analysis confirmed amplification specificity. Relative enrichment was calculated using the ΔCt method and normalized to input DNA, with data expressed as % input. Results represent mean ± standard deviation (SD) from three independent biological replicates.

### 2.4. Statistical Analysis

All statistical analyses were performed in R version 4.4.2 (R Foundation for Statistical Computing, Vienna, Austria). Associations between ChIP–qPCR enrichment (% recovery) and relative gene expression were evaluated separately for each gene, genomic region, and histone mark using Spearman’s rank correlation coefficient (ρ), as a non-parametric approach suitable for small sample sizes and potential non-linear relationships. For each analysis, the number of complete paired observations, correlation coefficient, and corresponding *p*-value were calculated. *p*-values were adjusted for multiple testing using the Benjamini–Hochberg false discovery rate (FDR) procedure across all correlation tests. Correlation analyses were conducted only when at least three complete paired observations were available and when variability was present in both variables

## 3. Results

### 3.1. Verification of Adipogenic Differentiation by BODIPY Staining

**Figure 1:**
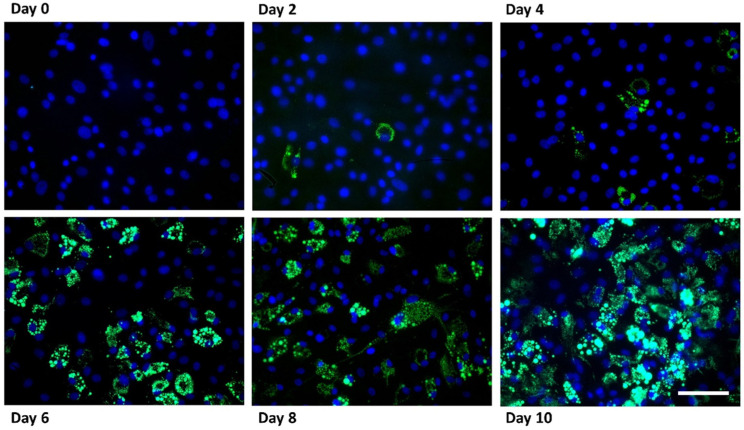
BODIPY staining during adipogenic differentiation of porcine mesenchymal stem cells. Representative fluorescence images showing intracellular lipid droplet accumulation at different stages of adipogenic differentiation (day 0, 2, 4, 6, 8 and 10). Lipid droplets are stained with BODIPY (green) and nuclei are counterstained with DAPI (blue). Scale bar: 50 µm.

Adipogenic differentiation of porcine mesenchymal stem cells was verified by BODIPY staining, which enabled visualization of intracellular lipid droplets during the differentiation process (Figure 1). Minimal lipid accumulation was observed at day 0, whereas small lipid droplets became detectable at day 2 and day 4. A marked increase in lipid droplet formation was observed at later stages of differentiation, particularly from day 6 onwards. By day 8 and day 10, extensive lipid accumulation was evident in the majority of cells (Figure S1). These observations confirmed successful adipogenic differentiation under the applied culture conditions.

### 3.2. Genomic Location of the Studied Genes

**Figure 2:**
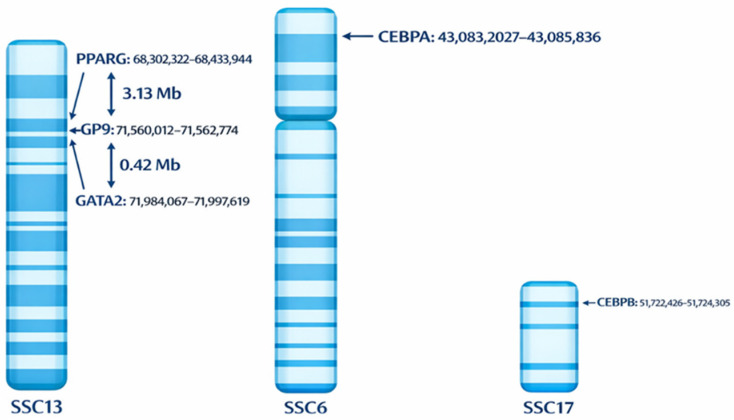
Schematic representation of the genomic locations of and *PPARG, GP9* and *GATA2* on chromosome 13 (SSC13), *CEBPA* on chromosome 6 (SSC6) and *CEBPB* on chromosome 17 (SSC17) (Sscrofa11.1). The diagram illustrates the chromosomal spacing between loci to highlight their genomic positions.

In *Sus scrofa*, the genomic organization of the analyzed genes demonstrates substantial diversity in both chromosomal location and gene size. The *PPARG* gene is positioned on chromosome 13 at chr13: 68,302,322–68,433,944, spanning 131,622 bp. On the same chromosome, the negative regulator *GATA2* is located at chr13: 71,984,067–71,997,619 with a total length of 13,552 bp, whereas the non-adipogenic control gene *GP9* resides nearby at chr13: 71,560,012–71,562,774 and spans 2762 bp (Sscrofa11.1 assembly). *CEBPA* is positioned on chromosome 6 at chr6: 43,083,207–43,085,836, spanning 2630 bp, while *CEBPB* resides on chromosome 17 at chr17: 51,722,426–51,724,305, spanning 1880 bp (Sscrofa11.1 assembly) (Figure 2). These genomic coordinates were retrieved from the Ensembl database (Sscrofa11.1 assembly). The heterogeneity in chromosomal positioning, gene size, and functional roles among these loci provides a strong rationale for investigating their region-specific chromatin states and histone modification dynamics during adipogenesis.

### 3.3. Dynamics of Gene Expression and Histone Modifications of PPARG and GATA2 During Adipogenesis

**Figure 3:**
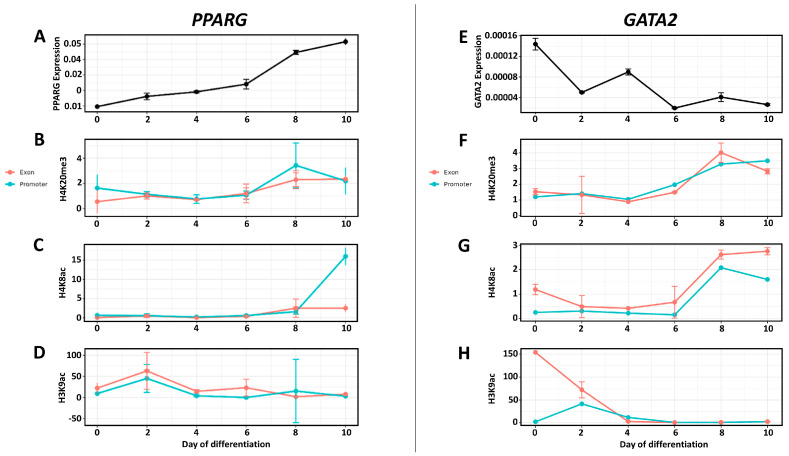
Gene expression and histone modifications of *PPARG* and *GATA2* during adipogenic differentiation. Relative mRNA expression and histone mark enrichment across differentiation stages (day 0, 2, 4, 6, 8, and 10) in porcine mesenchymal stem cells. The left panels correspond to *PPARG*, while the right panels correspond to *GATA2*. (**A**,**E**) Relative gene expression determined by RT-qPCR. (**B**,**F**) H4K20me3 enrichment at promoter and exon regions. (**C**,**G**) H4K8ac enrichment at promoter and exon regions. (**D**,**H**) H3K9ac enrichment at promoter and exon regions. Histone mark enrichment was measured by ChIP-qPCR and expressed as a percentage of input DNA. Data are presented as mean ± SD of biological replicates.

*PPARG* expression demonstrated a clear temporal increase throughout adipogenic differentiation (Figure 3A). The transcription levels were low at day 0, followed by a gradual elevation at days 2 and 4. A more pronounced upregulation was detected at day 6, with maximal transcript abundance observed at day 8 and day 10.

Analysis of histone modifications revealed dynamic changes at both promoter and exon regions of the *PPARG* locus. H4K20me3 enrichment remained low and relatively stable from day 0 to day 6 in both regions (Figure 3B). Beginning at day 8, enrichment levels increased, with the higher value observed for promoter site. H4K8ac enrichment was very low during early and intermediate stages (day 0–day 6), and the profiles were comparable in both promoter and exon regions (Figure 3C). A marked increase was observed at day 8, which persisted or further increased at day 10 in the promoter region. H3K9ac displayed a high level compared to the 2 previous modifications (Figure 3D). An early enrichment peak was observed at day 2 in both promoter and exon regions. Following this peak, enrichment decreased. Overall, the *PPARG* locus exhibited stage-dependent alterations in activating (H4K8ac, H3K9ac) and repressive (H4K20me3) histone marks across promoter and exon regions, with the most pronounced changes observed for histone acetylation.

*GATA2* expression was highest at day 0 and decreased markedly following induction of adipogenic differentiation (Figure 3E). A pronounced reduction was observed at day 2. Although a transient increase was detected at day 4, expression levels remained lower than baseline and continued at relatively reduced levels to the end of differentiation. Analysis of histone modification enrichment revealed stage-dependent alterations at both promoter and exon regions of the *GATA2* locus. H4K20me3 enrichment remained low and relatively stable from day 0 to day 6 in both regions (Figure 3F). An increase was observed at day 8 and day 10, with higher enrichment levels detected at the promoter compared to the exon at late stages. H4K8ac levels were generally low during early differentiation stages (Figure 3G). A moderate increase was observed at day 8 and day 10, particularly in the exon region. H3K9ac displayed elevated enrichment at day 0 in the exon region, followed by a substantial decrease beginning at day 2 (Figure 3H). H3K9ac levels in the promoter remained comparatively low throughout differentiation, with a peak at day 2.

To determine whether chromatin remodeling patterns observed at adipogenic transcription factor loci were gene-specific, histone modification enrichment was also examined at the *GP9* locus, a non-adipogenic gene located on the same chromosome as *PPARG* and *GATA2*. H4K20me3 enrichment displayed dynamic variation across differentiation stages (Figure S2A), with elevated levels at early stages and a pronounced peak at day 4, particularly in the exon region. Enrichment decreased substantially at later stages (day 6–day 8) before partially increasing again at day 10. H4K8ac levels showed moderate enrichment across early and intermediate stages in both promoter and exon regions (Figure S2B), followed by a decline at day 10. In contrast, H3K9ac enrichment was highest at day 0, particularly in the exon region, and decreased sharply thereafter, while promoter-associated enrichment remained low throughout differentiation (Figure S2C). Overall, the *GP9* locus exhibited temporal variation in histone modification enrichment during adipogenic differentiation. These results indicate that although the three genes (*PPARG, GP9* and *GATA2*) are located close to each other, each exhibits a distinct histone modification profile.

Taken together, in *PPARG* and *GATA2* genes, variable histone acetylation patterns was observed in the promoter and exon regions, suggesting that this modification may act as a regulatory component of these genes. In contrast, H4K20me3 did not show a clear correlation with gene expression, and changes in this repressive modification appear to be less directly associated with transcription levels.

### 3.4. Dynamics of Gene Expression and Histone Modifications of CEBPA and CEBPB During Adipogenesis

**Figure 4:**
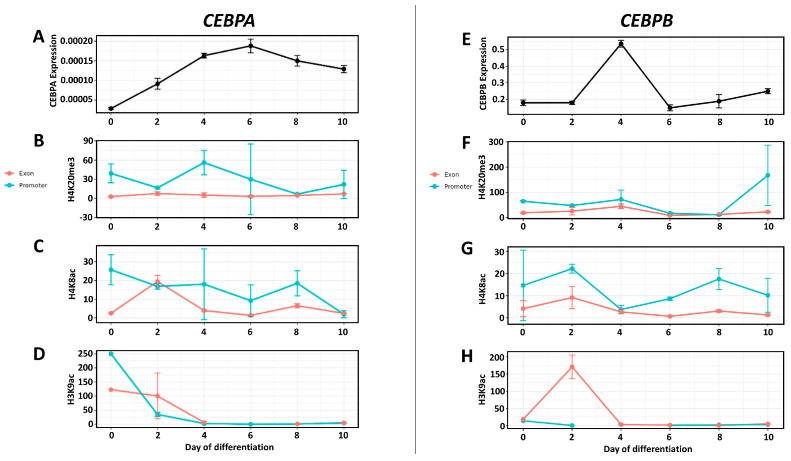
Gene expression and histone modification dynamics of *CEBPA* and *CEBPB* during adipogenic differentiation. Relative mRNA expression and histone mark enrichment across differentiation stages (day 0, 2, 4, 6, 8, and 10) in porcine mesenchymal stem cells. The left panels correspond to *CEBPA*, while the right panels correspond to *CEBPB*. (**A**,**E**) Relative gene expression determined by RT-qPCR. (**B**,**F**) H4K20me3 enrichment at promoter and exon regions. (**C**,**G**) H4K8ac enrichment at promoter and exon regions. (**D**,**H**) H3K9ac enrichment at promoter and exon regions. Histone mark enrichment was measured by ChIP-qPCR and expressed as a percentage of input DNA. Data are presented as mean ± SD of biological replicates.

*CEBPA* expression displayed a distinct temporal profile during adipogenic differentiation (Figure 4A). Expression levels gradually increased from day 0 to day 4, followed by a more pronounced elevation at day 6, where peak transcript abundance was observed. After day 6, expression levels declined at day 8 and day 10, although remaining above baseline levels detected at day 0.

Histone modification analysis revealed dynamic and region-specific changes across the *CEBPA* locus. H4K20me3 enrichment showed a sharp increase at the promoter region on day 4 (Figure 4B). Outside of this time point, promoter enrichment levels remained low throughout the differentiation stages. In contrast, exon-associated H4K20me3 levels exhibited minimal variation over time. H4K8ac enrichment at the exon was elevated on day 2, followed by a gradual decline (Figure 4C). In comparison, H4K8ac levels at the promoter were generally lower and displayed less pronounced temporal variation than those observed in the exon region. H3K9ac enrichment was highest at day 0, particularly in the promoter region, and markedly decreased by day 2 (Figure 4D). From day 4 onward, low levels were maintained in both the promoter and exon regions throughout the differentiation process.

*CEBPB* expression exhibited a transient and dynamic pattern during adipogenic differentiation (Figure 4E). Expression levels were low at day 0 and day 2, followed by a marked increase at day 4. After reaching this peak, transcript levels declined at day 6 and remained at lower levels at day 8 and day 10. Histone modification profiling demonstrated stage-specific and region-dependent changes at the *CEBPB* locus. H4K20me3 enrichment remained relatively stable and comparable for both studied genomic regions throughout the differentiation timeline, with only a peak at day 10 for promoter (Figure 4F). H4K8ac enrichment showed comparable profile for both exon and promoter, with small fluctuations more visible for promoter (Figure 4G). The level of this modification was low compared to the other two. H3K9ac demonstrated a distinct enrichment pattern in the exon region, with a marked peak at day 2 (Figure 4H). Following this early increase, enrichment levels declined and remained low across subsequent differentiation stages. Promoter H3K9ac levels remained minimal throughout the time course.

A summary of these results suggests that increases in H4K8ac and/or H3K9ac occur before or coincide with increases in gene expression, indicating that histone acetylation may prime chromatin for transcriptional activation. At many time points, the promoter region exhibits higher levels of acetylation than the exon region, suggesting that epigenetic regulation occurs primarily at the promoter level. In contrast, H4K20me3 does not show a clear correlation with gene expression levels, and its changes appear to be less closely associated with transcriptional activity than those of histone acetylation.

### 3.5. Correlation Between Histone Mark Enrichment and Gene Expression

Spearman’s rank correlation analysis was performed to evaluate the association between histone mark enrichment and gene expression levels across differentiation stages. To account for multiple testing, *p*-values were adjusted using the Benjamini–Hochberg false discovery rate (FDR) procedure. Overall, although several strong monotonic trends were observed, none of the correlations reached statistical significance after multiple testing correction (all FDR ≥ 0.200). Therefore, these results should be interpreted with caution and are considered exploratory. A comprehensive overview of all correlation coefficients and corresponding FDR-adjusted *p*-values is provided in the Supplementary Materials (Figure S3). In general, stronger correlations were observed in exon regions than in promoter regions, which may suggest a potential role of the analyzed histone modifications in regulating chromatin structure within the gene body.

## 4. Discussion

Adipose tissue differentiation is a complex biological process characterized by dynamic chromatin remodeling and tightly regulated transcriptional events. In this study, we investigated temporal patterns of histone modifications in the promoter and exon regions of four key adipogenic transcription factors in porcine mesenchymal stem cells and evaluated their association with gene expression during differentiation. Histone modifications constitute a major epigenetic mechanism regulating chromatin accessibility and transcriptional competence during cellular differentiation. In adipogenesis, activating histone marks, such as acetylation, are generally associated with open chromatin and transcriptional activation, whereas repressive methylation marks are linked to chromatin compaction and transcriptional silencing. Therefore, dynamic changes in these modifications are expected to contribute to the coordinated activation of pro-adipogenic genes and repression of anti-adipogenic regulators [1,13,16].

Our results demonstrated that histone modifications are dynamically regulated over time during adipogenesis, exhibiting gene-specific temporal patterns. The observed differences in the timing and rate of these changes suggest that epigenetic regulation is tightly coordinated with the progression of the differentiation program and may contribute to stage-specific gene expression. The progressive upregulation of *PPARG* observed in our model is consistent with its established role as a central regulator of terminal adipocyte differentiation [1,23]. Previous genome-wide studies in murine and human systems have demonstrated increased acetylation at the *PPARG* locus during adipogenesis, particularly enrichment of H3K9ac and H3K27ac at regulatory regions [11,24]. In agreement with these findings, we observed late-stage elevation of H4K8ac at both promoter and exon regions of *PPARG*, coinciding temporally with maximal transcript levels. Our study highlights the importance of H4K8ac enrichment in porcine adipogenesis through gene- and region-specific analysis. *GATA2*, known to function as an anti-adipogenic factor that must be downregulated for adipocyte commitment [17,25], displayed declining expression following induction of differentiation. This transcriptional repression was accompanied by reduced H3K9ac enrichment at early stages and increased H4K20me3 at later time points, suggesting a shift toward a more repressive chromatin state. Similar epigenetic transitions have been reported in murine adipogenesis models, where loss of activating acetylation marks at anti-adipogenic loci facilitates lineage progression [1,16]. An additional point of interest in the present study is that *PPARG* and *GATA2* are located on the same porcine chromosome, yet they displayed clearly distinct temporal patterns of histone modification during adipogenesis. This observation suggests that chromosomal proximity alone does not impose uniform epigenetic regulation, and that local chromatin context may be more relevant than linear genomic distance. This may reflect locus-specific regulatory features, such as differences in chromatin accessibility, transcription factor binding, or local regulatory element activity. Consequently, genes located within the same chromosomal region may still undergo distinct epigenetic regulation depending on their local regulatory environment. However, no direct chromatin conformation data were generated in this study, and therefore this interpretation requires further validation. This issue is particularly important in pigs, as although many adipogenesis-related genes are evolutionarily conserved, their chromosomal localization differs between pig and human, which may influence higher-order chromatin organization and regulatory interactions [26]. *CEBPA* exhibited peak expression at intermediate stages, in agreement with its established role in consolidating adipocyte identity [27]. The region-specific nature of this association underscores the importance of distinguishing promoter and exon chromatin states, as gene body and promoter acetylation may reflect distinct regulatory processes [28]. The transient induction of *CEBPB* at early stages of differentiation aligns with its established function as an early adipogenic initiator [29]. Correspondingly, we observed early promoter-associated acetylation, particularly H4K8ac enrichment at day 2, preceding peak transcript levels. This pattern is consistent with reports that *CEBPB* activation is accompanied by rapid chromatin remodeling during mitotic clonal expansion in 3T3-L1 cells [30].

Our analyses indicate that histone modification dynamics are not uniformly distributed across gene regions at the examined loci. This interpretation is supported by the observation that promoter- and exon-associated enrichment patterns frequently differed in both magnitude and temporal trajectory. Such region-specific behavior is consistent with genome-wide chromatin studies demonstrating functional compartmentalization of histone modifications between regulatory and coding regions [12,28]. The distinction between promoter- and gene body-associated histone modifications is particularly relevant in the context of transcriptional regulation. Promoter-associated acetylation is often linked to transcriptional initiation and recruitment of regulatory complexes, whereas histone marks within gene bodies may reflect transcriptional elongation, chromatin accessibility, or broader structural organization of active loci. In this context, the differences observed between promoter and exon regions in our study support the view that region-specific chromatin profiling provides more informative insight than gene-level analysis alone [1,12,28]. This spatial heterogeneity suggests that promoter-centered chromatin remodeling may play a primary role in transcriptional initiation, whereas exon-associated marks may reflect transcriptional elongation or broader chromatin organization. The temporal differences observed between promoter- and exon-associated histone modifications suggest that distinct chromatin regions may be differentially regulated at specific stages of adipogenesis, contributing to the precise coordination of gene expression programs during differentiation.

Beyond linear gene organization, chromatin is arranged in three-dimensional structures that include loops bringing distal regulatory elements into spatial proximity with promoters. These chromatin loops may span from kilobases to megabases and are dynamically reorganized during differentiation. Histone modifications are thought to contribute to loop formation and stabilization by altering chromatin accessibility and nucleosome organization [31,32]. Therefore, if genes located on the same chromosome undergo different spatial repositioning during adipogenesis, they may also acquire distinct local histone modification patterns. This may help explain why *PPARG* and *GATA2*, despite their chromosomal proximity, exhibited different epigenetic profiles in the present study [26,33,34]. The inclusion of *GP9* as a non-adipogenic locus located on the same chromosome as *PPARG* and *GATA2* enabled a partial assessment of genomic context effects. Although temporal variation in histone mark enrichment was observed at the *GP9* locus, the absence of transcriptional profiling prevented evaluation of expression–chromatin coupling. Nevertheless, the presence of chromatin dynamics at this locus suggests that differentiation-associated epigenetic remodeling may extend beyond strictly adipogenic genes.

Our findings should also be interpreted in the context of previous porcine studies on adipogenic epigenetic regulation. Stachecka et al. [10] demonstrated dynamic alterations in active and repressive histone marks at the protein level during adipogenic differentiation of porcine mesenchymal stem cells using Western blot analysis. Although our study assessed locus-specific enrichment by ChIP-qPCR rather than global protein abundance, both datasets support the view that adipogenesis in porcine cells is accompanied by coordinated remodeling of activating and repressive histone signatures. In addition, Kociucka et al. [35] reported correlations between histone modifications and expression of key adipocyte-related genes in porcine adipose tissue, further supporting the biological relevance of histone-mediated regulation in pigs [10,35]. Therefore, while temporal concordance between chromatin modification and transcriptional activity was observed, our findings should be interpreted as descriptive of dynamic association rather than conclusive evidence of direct regulatory coupling. It is also worth noting that epigenetic regulation of adipogenesis may be influenced by environmental and nutritional factors. In particular, nutritional compounds capable of modulating histone deacetylase activity may affect adipocyte differentiation and lipid accumulation. In porcine adipose tissue, short-chain fatty acids have been shown to enhance adipocyte differentiation, highlighting the possibility that metabolic inputs may interact with chromatin-level regulation during adipogenesis [36].

Future studies employing genome-wide epigenomic profiling approaches and increased temporal resolution will be necessary to more precisely define chromatin–transcription relationships during porcine adipogenesis. In particular, integration of genome-wide chromatin profiling techniques such as ChIP-seq or ATAC-seq would enable the identification of additional regulatory elements and facilitate evaluation of whether comparable epigenetic patterns are conserved across species [37,38]. Moreover, emerging methods for analysis of higher-order chromatin architecture and chromatin conformation [39,40] may provide further insight into how three-dimensional genome organization contributes to adipogenic gene regulation.

## 5. Conclusions

This study provides insight into histone modifications at key adipogenic transcription factor genes during the differentiation of porcine mesenchymal stem cells. By integrating gene expression profiling with promoter- and exon-specific analyses of histone marks, we identified dynamic, stage-dependent changes in activating (H3K9ac, H4K8ac) and repressive (H4K20me3) histone modifications at adipogenic loci. The temporal patterns of histone modifications only partially paralleled transcriptional dynamics, indicating complex chromatin remodeling throughout differentiation. Notably, histone modification dynamics were frequently region-specific, with promoter and exon regions displaying distinct enrichment trajectories. Overall, our findings support a model in which adipogenic differentiation is accompanied by coordinated, yet gene- and region-specific, chromatin remodeling.

## Data Availability

The data supporting the findings of the study are available from the corresponding author upon reasonable request.

## Supplementary Materials

The following supporting information can be downloaded at: https://www.mdpi.com/article/10.3390/genes17050521/s1. Table S1. Primer sequences, amplicon lengths, and annealing temperatures used for qPCR and ChIP-qPCR amplification of promoter and exon regions of target genes. Table S2. Antibodies used for ChIP-qPCR, including target specificity, host species, provider information, catalog numbers, and amounts applied per immunoprecipitation. Figure S1. Lipid accumulation during adipogenic differentiation assessed by BODIPY fluorescence intensity. A gradual increase in lipid accumulation was observed over time, with a marked elevation from day 6 onwards and a peak at day 10. Data represent mean fluorescence intensity values corresponding to each time point. Figure S2. Histone modification profiles at the GP9 locus during adipogenic differentiation. Enrichment of H4K20me3 (A), H4K8ac (B), and H3K9ac (C) at promoter and exon regions of the GP9 locus across six differentiation stages (from day 0 to day 10) in porcine mesenchymal stem cells. Histone mark enrichment was determined by ChIP-qPCR and expressed as percentage of input DNA. Figure S3. Spearman correlation analysis between histone mark enrichment and gene expression. Heatmap representation of Spearman correlation coefficients (ρ) between histone mark enrichment (H4K20me3, H4K8ac, and H3K9ac) at promoter and exon regions and corresponding gene expression levels across differentiation stages. Correlation coefficients and FDR-adjusted *p*-values (Benjamini–Hochberg correction) are indicated within each cell. No correlations reached statistical significance after multiple testing correction.
