# Supplementary material for: Dynamic Histone Modification Patterns in Key Transcription Factor Genes During Porcine Adipogenesis
Source: Genes (Basel). 2026 Apr 28;17(5):521. doi: 10.3390/genes17050521 (PMC13205497; doi:10.3390/genes17050521)
Supplement: Supplementary file 1 [file genes-17-00521-s001.zip › genes-4250770-supplementary.pdf]

Supplementary material

Table S1. Primer sequences, amplicon lengths, and annealing temperatures used for qPCR and ChIP-qPCR amplification of promoter and exon regions of target genes.

**Primer Names**	**Sequences**	**Amplicon length**	**Annealing Temprature**
*CEBPA*_Exon	F: 5` CGTGAGCGCAACAACATCG 3`<br>R: 5` CTCAGTTGTTCCACCCGCTT 3`	131 bp	60°C
*CEBPA*_Promoter	F: 5’ TGGGGAAGCCAGAGGAGAG 3`<br>R: 5’ GTTAGAGGGCGGTGAGTGG 3`	181 bp	60°C
*CEBPB*_Exon	F: 5` TACTACGAGGCGGACTGCTT 3`<br>R: 5` TCCAGGTATGGGCTGAAGTC 3`	152 bp	60°C
*CEBPB*_Promoter	F: 5’ AAAGCCCCCAACCACAAGTC 3`<br>R: 5’ TCAAGATCAGCGGCTCTCAG 3`	233 bp	60°C
*PPARG*_Exon	F: 5’ GGATCAGCTCTGTGGACCTG 3`<br>R: 5’ GATCAGCTCTCGGGAATGGG 3`	132 bp	60°C
*PPARG*_Promoter	F: 5’ CAGGATGCGCTGGTGTTTG 3`<br>R: 5’ CTTCTGACCGAGCCTGACTC 3`	104 bp	60°C
GATA2 Exon	F: 5’ CACACTTGTTGCACAGCCC 3`<br>R: 5’ CTTGGAGAAGGGGTTGACGG 3`	110 bp	60°C
GATA2 Promoter	F: 5’ TCTGTGAACAGGCAGCAGTC 3`<br>R: 5’ ACCTTGGGCCCAAACAGAAA 3`	137 bp	60°C
GP9 Exon	F: 5’ GGGGCTGGAGGTGGACTG 3`<br>R: 5’ CAGAGGCGCAAGTACGTGAG 3`	201 bp	60°C
GP9 Promoter	F: 5’ TTCCAAGCTGACAAGTGCCA 3`<br>R: 5’ GCTCTCAGAACACCTGCCAT 3`	118 bp	60°C
RPL27	F: 5’ GCAAAGCGGTCATCGTAAA 3`<br>R: 5’ CTTGTGGGCATGAGGTGAT 3`	190 bp	60°C
PPIA	F:5’ CACAAACGGTTCCCAGTTTT 3`<br>R:5’ TGTCCACAGTCAGCAATGGT 3`	171 bp	60°C

 https://doi.org/10.3390/xxxxx

Table S2. Antibodies used for ChIP-qPCR, including target specificity, host species, provider information, catalog numbers, and amounts applied per immunoprecipitation.

Target Mark	Antibody name and Specifity	Host	Company	Cat. No.	Amount/IP	Notes
H4K20me3	Anti-Histone H4 (tri-methyl K20), ChIP-grade	Rabbit	Abcam	ab9053	2–5 µg	Polyclonal
H4K8ac	Anti-Histone H4 (acetyl K8), ChIP-grade	Rabbit	Abcam	ab15823	2–5 µg	Polyclonal
H3K9ac	Anti-Histone H3 (acetyl K9), ChIP-grade	Rabbit	Abcam	ab10812	2–5 µg	Polyclonal
IgG (Negative control)	Normal Rabbit IgG	Rabbit	Diagenode	Included in kit (Kit Cat# C01010051)	Matched volume	Provided in iDeal ChIP-seq Kit

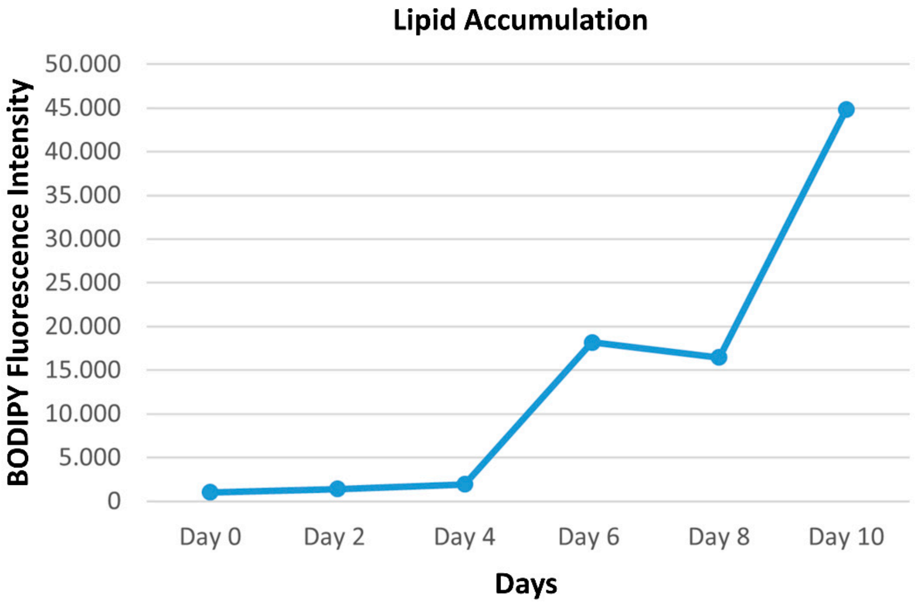

Figure S1. Lipid accumulation during adipogenic differentiation assessed by BODIPY fluorescence intensity. A gradual increase in lipid accumulation was observed over time, with a marked elevation from day 6 onwards and a peak at day 10. Data represent mean fluorescence intensity values corresponding to each time point.

https://doi.org/10.3390/xxxxx

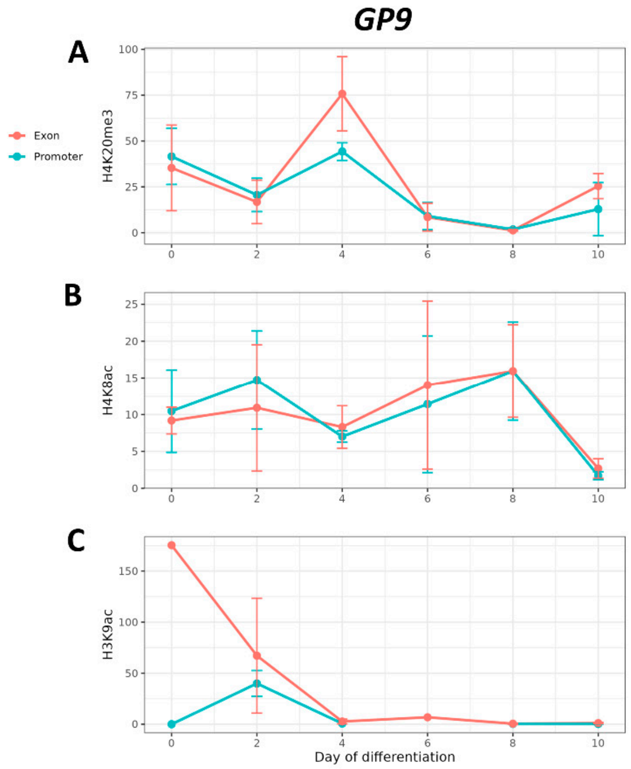

Figure S2. Histone modification profiles at the *GP9* locus during adipogenic differentiation. Enrichment of H4K20me3 (A), H4K8ac (B), and H3K9ac (C) at promoter and exon regions of the *GP9* locus across six differentiation stages (from day 0 to day 10) in porcine mesenchymal stem cells. Histone mark enrichment was determined by ChIP-qPCR and expressed as percentage of input DNA.

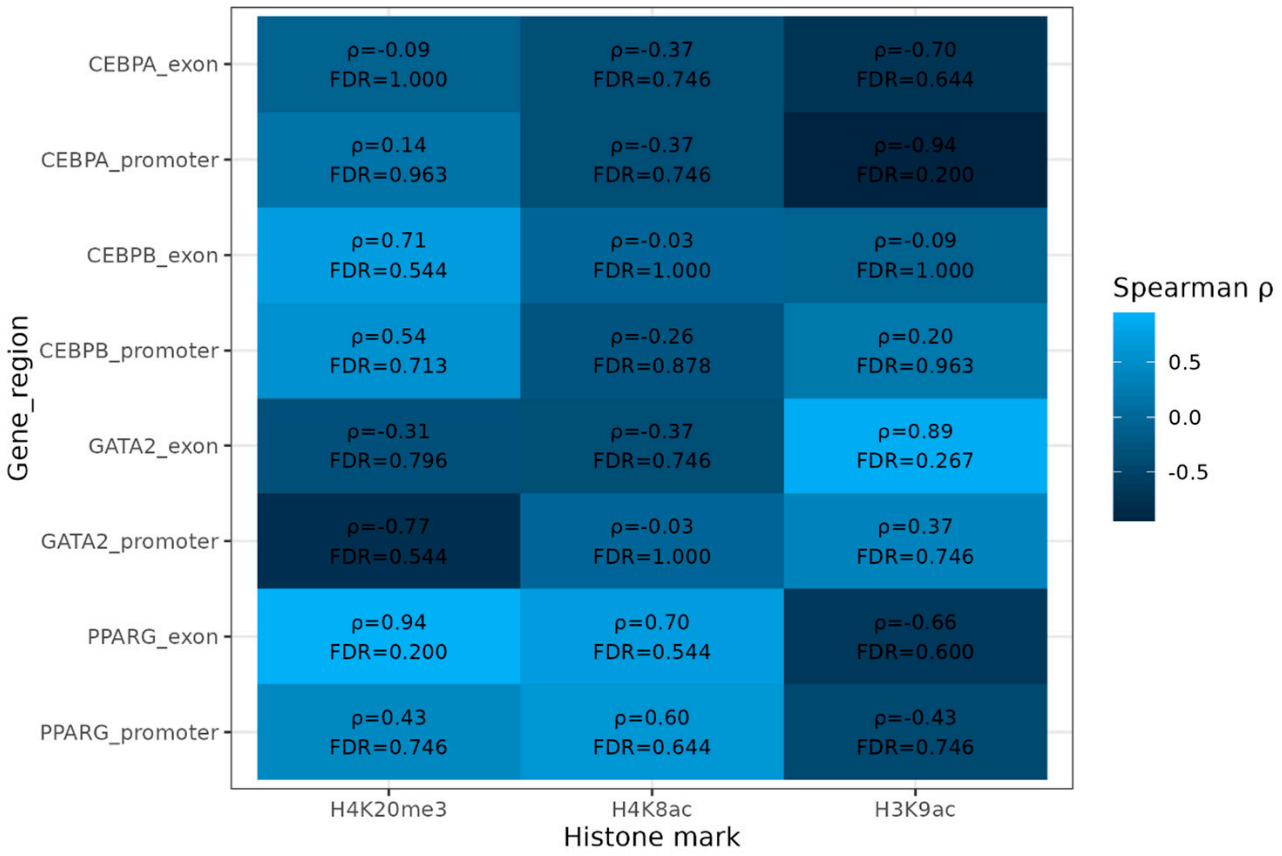

Figure S3. Spearman correlation analysis between histone mark enrichment and gene expression. Heatmap representation of Spearman correlation coefficients (ρ) between histone mark enrichment (H4K20me3, H4K8ac, and H3K9ac) at promoter and exon regions and corresponding gene expression levels across differentiation stages. Correlation coefficients and FDR-adjusted p-values (Benjamini–Hochberg correction) are indicated within each cell. No correlations reached statistical significance after multiple testing correction.
